# Response to Comment: Gas Bubbles from Biodegradable Magnesium Implants Convey Mechanical Cues and Promote Immune Cell Stimulation

**DOI:** 10.1002/advs.202520368

**Published:** 2025-11-25

**Authors:** Heithem Ben Amara, Jincy Philip, Omar Omar, Peter Thomsen

**Affiliations:** ^1^ Department of Biomaterials Institute of Clinical Sciences Sahlgrenska Academy University of Gothenburg Gothenburg SE‐405 30 Sweden; ^2^ Department of Biomedical Dental Sciences College of Dentistry Imam Abdulrahman bin Faisal University Dammam 31441 Saudi Arabia

## 1 Response

We are grateful to Guo et al.^[^ ^1^ ^]^ for their insightful commentary on our study.^[^ ^2^ ^]^ Their perspective contextualizes our findings within the broader landscape of biodegradable implant research and charts productive directions for future investigations. The commentary from Guo et al. rightly emphasizes two critical dimensions that warrant deeper examination: the precise chemical composition of gas bubbles generated during magnesium degradation, and the mechanical forces these bubbles exert on surrounding tissues. These considerations carry direct translational implications. On the one hand, defining quantitative thresholds for bubble accumulation could inform clinical monitoring strategies. On the other hand, harnessing mechanical gradients from bubbles may enhance magnesium implant integration. It is worth reflecting, however, on the remarkable biological complexity concentrated within the 20 µm‐width tissue territories we examined in our study, namely the narrow tissue interface surrounding bubbles or juxtaposing the implant. Within these spatially confined microenvironments, we observed a diverse cellular landscape comprising phagocytes with multiple, varied phenotypes, adaptive immune cells, stromal progenitors, and fibroblasts, each contributing to the orchestrated tissue response. Instead of viewing this interface as a mythical Pandora's box, we realize the complexity of material‐tissue biology, and it definitely spurs our curiosity and interest. This cellular heterogeneity within restricted spatial domains underscores both the precision required in analytical approaches and the multifaceted nature of host–biomaterial interactions that merit deeper consideration across methodological, translational, and clinical dimensions in the discussion that follows.

### 1.1 The Importance of the Spatial Dimension

Spatial transcriptomics represents a transformative shift in biomedical research.^[^ ^3^, ^4^ ^]^ In oncology and neuroscience, for instance, it has become instrumental in demonstrating that molecularly defined cell types acquire functional meaning only within their native spatial contexts.^[^ ^5^ ^]^ Neuronal transcriptional states manifest function through precise cortical coordinates,^[^ ^6^ ^]^ while tumor microenvironments display signaling gradients that drive oncogenic progression.^[^ ^7^ ^]^ Despite these advances across biomedical disciplines, spatial transcriptomic interrogation at implant–tissue interfaces remains notably nascent. This underutilization represents both a methodological gap and an opportunity to advance the understanding of host–biomaterial interactions via the lens of spatially resolved approaches.

The compelling rationale for integrating spatial dimensions into biomaterial research stems from a fundamental principle: biology at the implant–cell/tissue interface unfolds in space. Where cells encounter the implant surface, spatial position becomes a critical determinant of cellular response. Understanding this biology requires, in fact, resolving a multidimensional problem: identifying which cells respond, determining their functional programs, establishing temporal dynamics, and crucially, defining where these interactions occur.^[^ ^8^ ^]^ While multiomics platforms resolve cellular identities and functions, and longitudinal designs address timing, spatial assays uniquely define the site of implant–cell interactions. Bringing the spatial dimension may uncover the biology of the implant–cell/tissue interface that bulk approaches obscure.

Recent investigations have begun to demonstrate the value of spatially resolved analysis in biomaterial contexts.^[^ ^9^ ^]^ Bone regeneration studies^[^ ^10^ ^]^ integrating spatial transcriptomics with single‐cell RNA sequencing revealed distinct spatial preferences among mesenchymal stem cell sub‐clusters during bioactive implant‐assisted repair of calvarial defects. Notably, matrix Gla protein‐expressing osteoprogenitors localized into defect regions, while mature osteoblasts concentrated in regenerated bone tissue. These findings suggest that functionally distinct progenitor subsets occupy specific spatial niches during peri‐implant regeneration.

### 1.2 Why do Material–Tissue Interfaces Provide Technical Challenges for Spatial Omics?

Analysis of the implant–tissue interface presents a fundamental methodological challenge:^[^ ^11^ ^]^ sample processing may spatially alter the interfacial relationships, thus raising the critical question of whether we characterize authentic implant–tissue interfaces or preparation artefacts. Chemical fixation induces tissue shrinkage that creates artifactual gaps at the tissue‐implant boundary,^[^ ^12^ ^]^ whilst paraffin embedding necessitates metallic implant removal, thereby distorting the intact interface. Moreover, for biodegradable metallic implants, decalcification procedures dissolve both mineralized tissue and the adjoining metal, further altering the interfacial region.

Embedding in synthetic resins may partially overcome these issues by enabling sectioning of intact interfaces without implant removal. This permits the preservation of spatial relationships between the implant surface and adherent biological tissues.^[^ ^13^ ^]^ However, conventional resins polymerize at high temperatures, therefore denaturing DNA and proteins and precluding immunohistochemical or in situ hybridization techniques. Low‐temperature polymerizing resins address this limitation by enabling sectioning of intact interfaces, whilst preserving molecular targets.^[^ ^14^ ^]^ More significantly, emerging protocols for deplastification of methyl methacrylate‐embedded sections permit transcriptomic and proteomic on structurally intact interfaces.^[^ ^15^ ^]^ This convergence of advanced embedding techniques with spatial omics platforms opens avenues for multimodal correlative approaches that integrate molecular, cellular, and structural data from the same interface region, and may fundamentally transform the interrogation of biomaterial–tissue interactions without artifactual disruption.

Building upon these methodological advances, future investigations of bubble‐adjacent tissues should extend beyond transcriptomics to capture protein‐level regulation.^[^ ^16^ ^]^ Spatial proteomics, recognized as *Method of the Year 2024*,^[^ ^17^ ^]^ now enables antibody‐based multiplexed imaging (e.g., sequential immunofluorescence (SeqIF), co‐detection by indexing(CODEX), multiplexed ion beam imaging(MIBI), imaging mass cytometry(IMC)) and emerging deep visual proteomics that retain spatial context without antibody constraints.^[^ ^18^ ^]^

### 1.3 Are Current Biomaterial Treatments Truly Personalized?

It is pertinent to briefly discuss potential implications for biomaterials research of the techniques used in Ben Amara et al.^[^ ^2^ ^]^ and commented upon by Guo and coworkers.^[^ ^1^ ^]^ The route to treatment of patients with implants and prostheses follows multiple, rigorous steps, involving, for example, (bio)mechanical, toxicological, mutagenic, and biocompatibility standard assays alongside in vivo animal models and clinical trials.^[^ ^19^ ^]^ The important aim is to exclude potential adverse material and biological effects. However, these standardized assessments are typically conducted in healthy systems, whereas clinical populations present considerable heterogeneity. In the field of musculoskeletal implants, the personalization step is largely delegated to the surgeon who navigates systemic diseases, local tissue conditions, age, medications, smoking, and other circumstances that influence the decisions to be made pre‐ and post‐operatively. In dentistry and orthopedics, results of clinical trials and nation‐wide clinical quality registers^[^ ^20^ ^]^ influence such decisions. Now, given the excellent clinical results, for example, using oral implants,^[^ ^21^ ^]^ amputation prostheses^[^ ^22^ ^]^ and arthroplasties,^[^ ^23^ ^]^ it could be concluded that large patient cohorts have undeniably gained an improved quality of life at reasonable personal and societal costs. Nevertheless, the individual patient does not usually receive a tailor‐made biomaterial and adjunct treatment targeting precise structural, disease‐specific, or immune‐related molecular mechanisms decisive for the implant performance. It is apparent that large, major groups of patients with systemic diseases have local, specific unmet needs of treatment and rehabilitation. For example, patients with diabetes, osteoporosis, or having sequelae after radiation present a locally compromised situation where the host response is dysregulated, yet persistently treated with implants developed by evaluation in healthy tissues.

### 1.4 Connecting Spatial Knowledge with Personalized Biomaterials Therapy

As mentioned above, a revolution is ongoing in finding targets for treatments of specific cancers, neurological, and other diseases. Likewise, an important step is to gain fundamental knowledge of how the host handles the surgery in the presence of a material implanted in the body, irrespective if the local tissue is “*normal*” or compromised. Today, the ongoing research and development of sophisticated material manufacturing and surface modification techniques hold promise for promoting spatially and even time‐resolved biological effects. With an emerging detailed understanding of the cell function at the implant–tissue interface, an opportunity for truly personalized biomaterial therapies will emerge. This will require that both “*normal*” and compromised tissue responses are addressed with methodologies expanding beyond the traditional tools used in the path to acquire regulatory approval of a medical device. In turn, this would also require that the new, emerging spatial biology knowledge and techniques become disseminated and affordable for not only preclinical biomaterial scientists but also clinical researchers, contract research organizations, and medical device manufacturers. Ultimately, it is envisioned that the emerging knowledge will be an important driving force toward future treatments, especially for patients with locally compromised conditions requiring biomaterials.

## Conflict of Interest

The authors declare no conflict of interest.
